# Supplementary material for: High-Efficiency Water Recovery from Urine by Vacuum Membrane Distillation for Space Applications: Water Quality Improvement and Operation Stability
Source: Membranes (Basel). 2022 Jun 17;12(6):629. doi: 10.3390/membranes12060629 (PMC9230999; doi:10.3390/membranes12060629)
Supplement: Supplementary file 1 [file membranes-12-00629-s001.zip › membranes-1728901-supplementary.pdf]

Supplementary Information

High-efficiency water recovery from urine by vacuum membrane distillation for space applications: water quality improvement and operation stability

Fei Wang[1,2], Junfeng Liu[1], Da Li[1], Zheng Liu[3], Jie Zhang[1], Ping Ding[2], Litao Liu[2], Guochang Liu[3] and Yujie Feng[1]*

[1] School of Environment, Harbin Institute of Technology, No. 73 Huanghe Road, Nangang District, Harbin 150090, China

[2] National Key Laboratory of Human Factors Engineering, China Astronaut Research and Training Center, Beijing 100094, China

[3] The Institute of Seawater Desalination and Multipurpose Utilization, MNR(Tianjin), Tianjin 300192, China

Number of pages: 13

Number of figures: 6

Number of tables: 3

*Corresponding Author:

E-mail: yujief@hit.edu.cn (Yujie Feng）

S1 Compositions of and concentrations in ersatz urine

Table S1. Compositions of and concentrations in ersatz urine.

Composition	Concentration (mg/L)	Composition	Concentration (mg/L)
NaCl	10,000	$CaCl_2$	50
KCl	350	Urea	18,000
K_2SO_4	350	Sarcosine	1200
$MgSO_4$	740	Hippuric acid	800
$NaHCO_3$	100	Uric acid	500
K_3PO_4	3000	L-*tert*-leucine	250
NaH_2PO_4	40	Butanedioic acid	50
NH_3	500	Phenol	200

S2 Properties of the PP hollow fiber membrane

In VMD, the hydrostatic pressure must be less than the liquid entry pressure of water (LEP_W) to avoid membrane penetration by the feed stream [1]. The LEPw can be quantified by the Laplace equation (Eq. S1) [1].

$$\mathrm{LEP_w} = \frac{-2\tau\gamma_l}{r_{max}}cos\theta > P_{\mathrm{feed\ side}} - P_{\mathrm{pore}} \quad \text{(S1)}$$

where τ is the tortuosity factor of the pores, γ_l is the surface tension of the solution, θ is the contact angle between the solution and the membrane surface (which depends on the hydrophobicity of the membrane), and r_{max} is the largest pore size. $P_{\mathrm{feed\ side}}$ is the liquid pressure in the feed side and P_{pore} is the air pressure in the membrane pore. In this study, the LEP_w of the fabricated PP membrane reached 0.2 Mpa, which is available for VMD.

Table S2 shows the major properties of the PP hollow fiber membrane; including the diameter, porosity, and mean pore size.

Table S2. Properties of the PP hollow fiber membrane.

VMD membrane	Parameter
Outer diameter (μm)	620 ± 50
Membrane thickness (μm)	60 ± 5
Mean pore size (nm)	282 ± 19
Porosity (%)	55 ± 1
Liquid entry pressure of water (MPa)	0.2
Water contact angle (°)	105 ± 2
Air permeability ($m^3/m^2 \cdot h$, N_2, 0.01 MPa)	8.5 ± 0.7
Rejection rate by VMD of NaCl solution (%, 2 g/L NaCl)	99.9%

Figure S1 shows the surface morphology and microstructure of the produced PP hollow fiber membrane. The typical structure of the PP hollow fiber membrane pores resulted from lamellar separation when the stacked crystalline lamellar was stretched in the precursor films [2]. The crystalline lamellar was arranged normal to the axial direction and a large number of micropores were formed with stretched. In addition, there was a slightly higher porosity of the inner surface than that of the outer surface, which was due to the temperature difference between the inner surfaces and outer surfaces of the hollow fibers during the melt spinning [2, 3] .

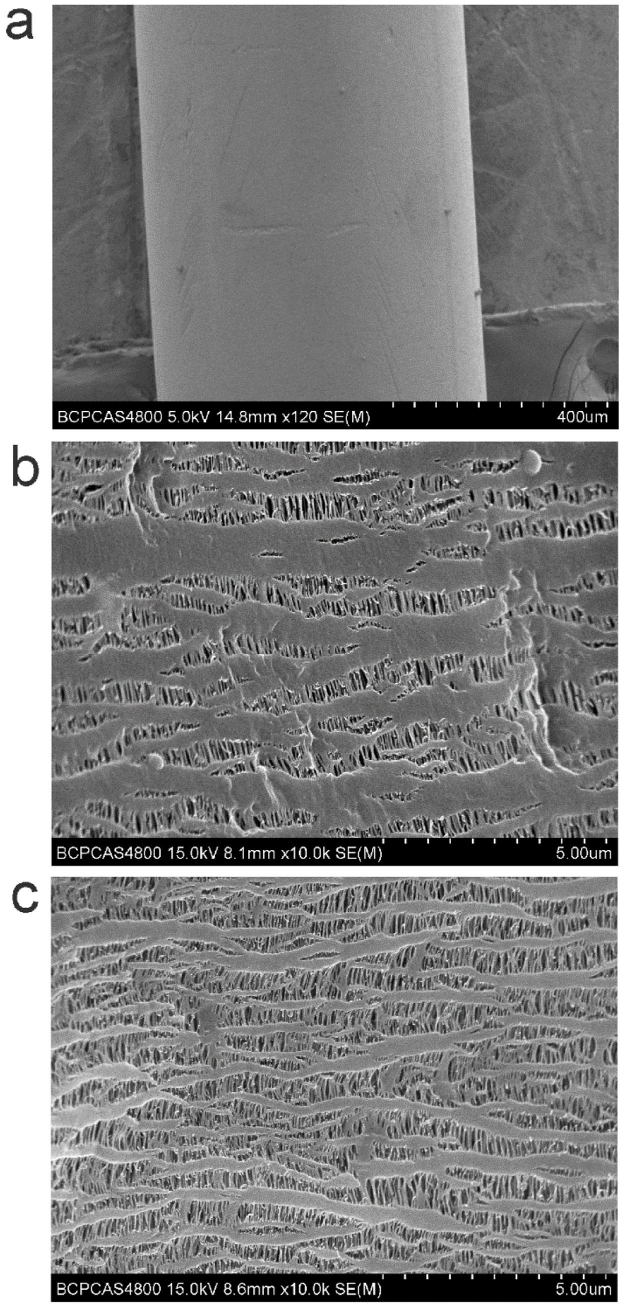

Figure S1. Surface morphology (a) and microstructure of the outer surface (b) and inner surface (c) of the PP hollow fiber membrane.

S3 Bacteria counts on spread plates for nonstabilized urine (a, b) and stabilized urine (c, d)

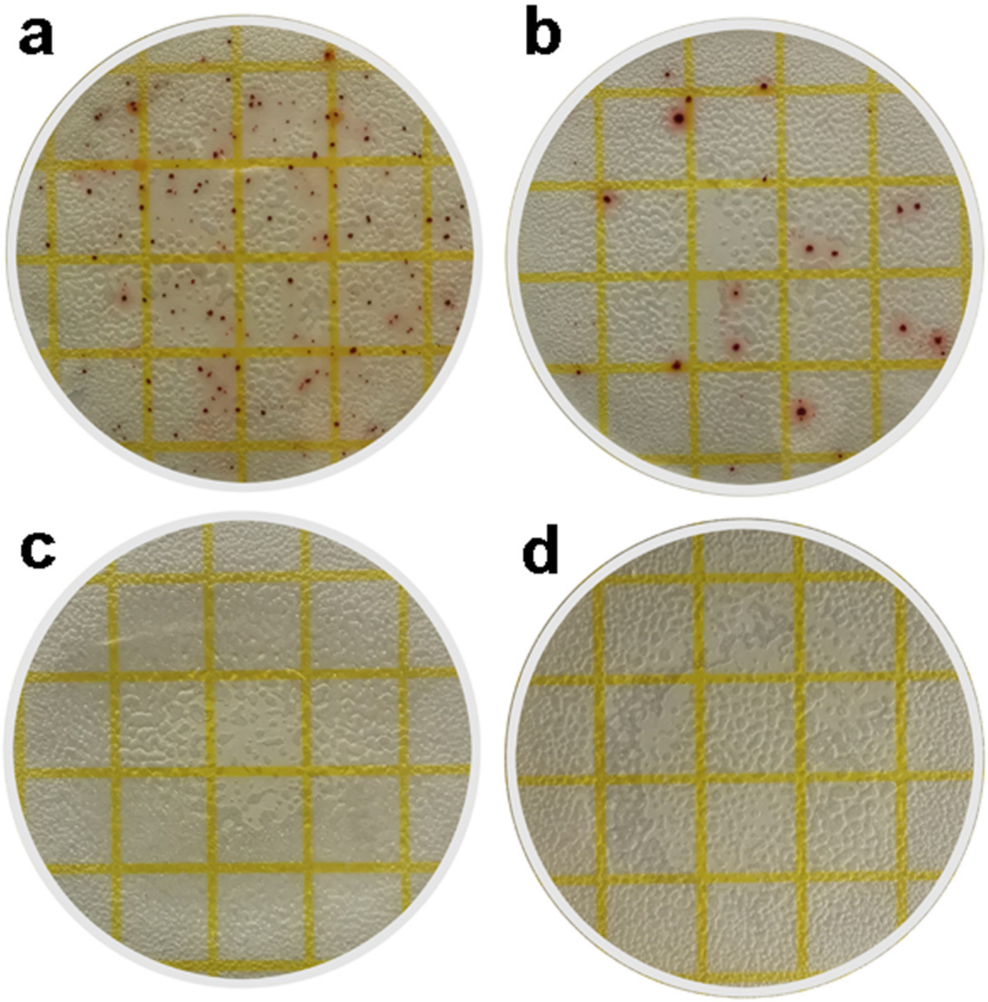

Figure S2. Bacteria counts on spread plates for nonstabilized urine (a, 1:1,000; b, 1:10,000) and stabilized urine (c, 1:1; d, 1:10).

S4 Basic theory of water recovery by VMD

The vapor flux through porous membranes is driven by the vapor pressure difference across the membrane during MD (Figure S3).

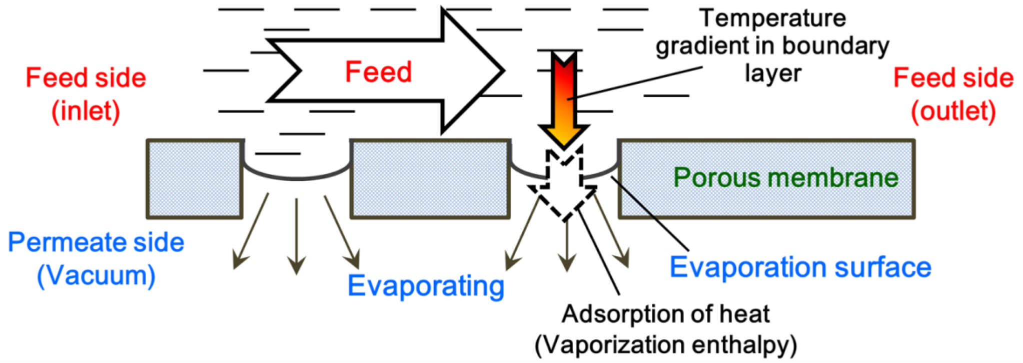

Figure S3. Heat transformation from the evaporation surface to the feed stream during VMD.

Three basic mechanisms dominate the vapor transport through the membrane pores: Knudsen diffusion (*K*), Poiseuille flow (*P*), and molecular-diffusion (*M*)—or a combination of these mechanisms, known as the transition mechanism [4]. However, for VMD the permeate side operates at a lower pressure than the vapor pressure of water, and only a trace quantity of gas is present in the membrane pores. Thus, the mass transfer resistance caused by molecule–molecule collision can be neglected. Therefore, the mass transfer across the membrane in VMD can be expressed by Eq. S2 [4].

$$N_{K-P} = N_K + N_P = \left[\frac{8}{3}\frac{\varepsilon r}{\tau\delta}\left(\frac{1}{2\pi RMT}\right)^{\frac{1}{2}} + \frac{r\varepsilon^2}{b\tau}\frac{1}{8\eta}\frac{P_m}{RT}\right]\Delta P \qquad \text{(S2)}$$

where $N_K = \frac{8}{3}\frac{\varepsilon r}{\tau\delta}\left(\frac{1}{2\pi RMT}\right)^{\frac{1}{2}}\Delta P$ and $N_P = \frac{r\varepsilon^2}{b\tau}\frac{1}{8\eta}\frac{P_m}{RT}\Delta P$ represent the contributions of Poiseuille flow and Knudsen diffusion to the mass transfer, respectively. In accordance with the kinetic theory of gases, when the mean free path of the water vapor molecules (λ) is large compared with the membrane pore size (d), the molecule–pore wall collisions are dominant over the Poiseuille flow; otherwise, the Poiseuille flow or Poiseuille flow–Knudsen transition mechanism dominates the mass transfer. During the mass transfer, the Knudsen number (Kn) is used to judge the dominant mechanism of the mass transfer in the pores [4]. When Kn > 1 the dominant mass transfer mechanism within the pore is Knudsen diffusion, whereas Kn < 0.01 and 0.01 < Kn < 1 represent the Poiseuille flow and transition mechanism, respectively [5, 6].

$$\mathrm{Kn} = \frac{\lambda}{d} \qquad \text{(S3)}$$

When the gas molecules are assumed to be hard spheres with diameter d_e and only binary collisions are involved, the average distance traveled by molecules to make collisions (λ) is defined by Eq. S4 [6].

$$\lambda = \frac{k_B T}{\sqrt{2}\pi P_m d_e^2} \quad \text{(S4)}$$

where K_B, T, and P_m are the Boltzman constant (1.38×10^{-23} J K^{-1}), temperature of the evaporation interface in the pores, and average pressure within the membrane pores [the average value of the vapor pressure (P_{vapor}) and permeate side pressure (P_{vacuum})], respectively [5]. The collision diameter, d_e, was ca. 2.64×10^{-10} m [5]. Because T was 55°C to 70°C and P_{vacuum} was 7.3–19.3 kPa, the λ calculated by Eq. S4 was ca. 0.61–1.27 μm; thus, Kn was ca. 2.18–4.54 when the mean pore diameter of the membrane was ca. 0.28 μm (Table S1). For Kn > 1, Knudsen diffusion dominated the mass transfer mechanism; thus, the mass transfer mechanism is expressed as follows [6].

$$N_{\mathrm{Kn}} = \frac{8}{3}\frac{\varepsilon r}{\tau\delta}\left(\frac{1}{2\pi RTM}\right)^{1/2}\Delta P \quad \text{(S5)}$$

where R is the universal gas constant, M is the molecular mass, r is the radius of the pore, δ is the thickness of the membrane across the membrane pores [$\delta = r_i \ln(r_o/r_i)$ for a hollow fiber membrane; were r_i and r_o are the inner and outer radius, respectively, of the hollow fiber], ε is the porosity of the membrane, and τ is the tortuosity factor of the pores. Because the evaporation interface temperature in the pores could not be measured directly, the mean values of the inlet and outlet temperatures of the feed (feed average temperature: $T_m = (T_i + T_o)/2$) were used for assessing the influence of the temperature on the permeate flux. P_{vapor} was determined by following Antoine's equation [1, 7]. P_{vacuum} is the absolute pressure of the permeate side.

$$P_{\mathrm{vapor}} = \exp\left(23.1964 - \frac{3816.44}{T_m - 46.13}\right) \quad \text{(S6)}$$

For a fixed membrane module, $\frac{8}{3}\frac{\varepsilon r}{\tau\delta}\left(\frac{1}{2\pi RM}\right)^{\frac{1}{2}}$ is constant, and the permeate flux in VMD varies with T and P_{vacuum}. When the VMD was operated at same temperature, the $P_{vapor,\,T}$ was a constant, and the function between N_{Kn} and P_{vacuum} can be expressed as a linear and character parameter a $= \left[\frac{8}{3}\frac{\varepsilon r}{\tau\delta}\left(\frac{1}{2\pi RT_m M}\right)^{\frac{1}{2}}\right]$, which was the slope of the $N_{Kn} - P_{vacuum}$ curves.

$$N_{\mathrm{Kn}} = \mathrm{a}\left(P_{vapor,T_m} - P_{vaccum}\right) \quad \text{(S7)}$$

S5 Ionization equilibrium of ammonia and phenol

Ammonia is in two forms in urine (volatile ammonia and ammonium ion), governed by the pH in accordance with the following chemical reaction [8].

$$NH_3 + H_2O \overset{K_1}{\leftrightarrow} NH_4^+ + OH^- \tag{S8}$$

where K_1 is the equilibrium constant for the reaction. When pH<5, 99.9% of the TAN is present as ammonium ions because the equilibrium of the reaction toward the right-hand side is ca 3.2×10^4 times higher than that of the left-hand side [8].

Phenol is in two forms in solution (phenol and phenolate ion). Herein, the concentration of phenol and phenolate ion was considered following Eq. S9 [9].

$$C_6H_5OH + OH^- \overset{K_2}{\leftrightarrow} C_6H_5O^- + H_2O \tag{S9}$$

According to a previous study [10], the dissociation constant (pK_2) is 9.55–9.97 over the temperature range of 25° to 60°C. Therefore, >99.99% of the phenol is present as molecules and ready to diffuse to the permeate side with water vapor at pH<2.

S6 pH and conductivity of phenol solution at 25 °C

Table S3. The pH and conductivity of phenol solution at 25 °C.

Phenol concentration (mg L^{-1})	Ph	Electrical conductivity (μs cm^{-1})
500	6.104	18.55
200	6.220	8.10
100	6.512	4.49
50	6.805	2.77
25	6.810	1.86

S7 Morphological and elemental analyses of membrane samples after 91% water recovery.

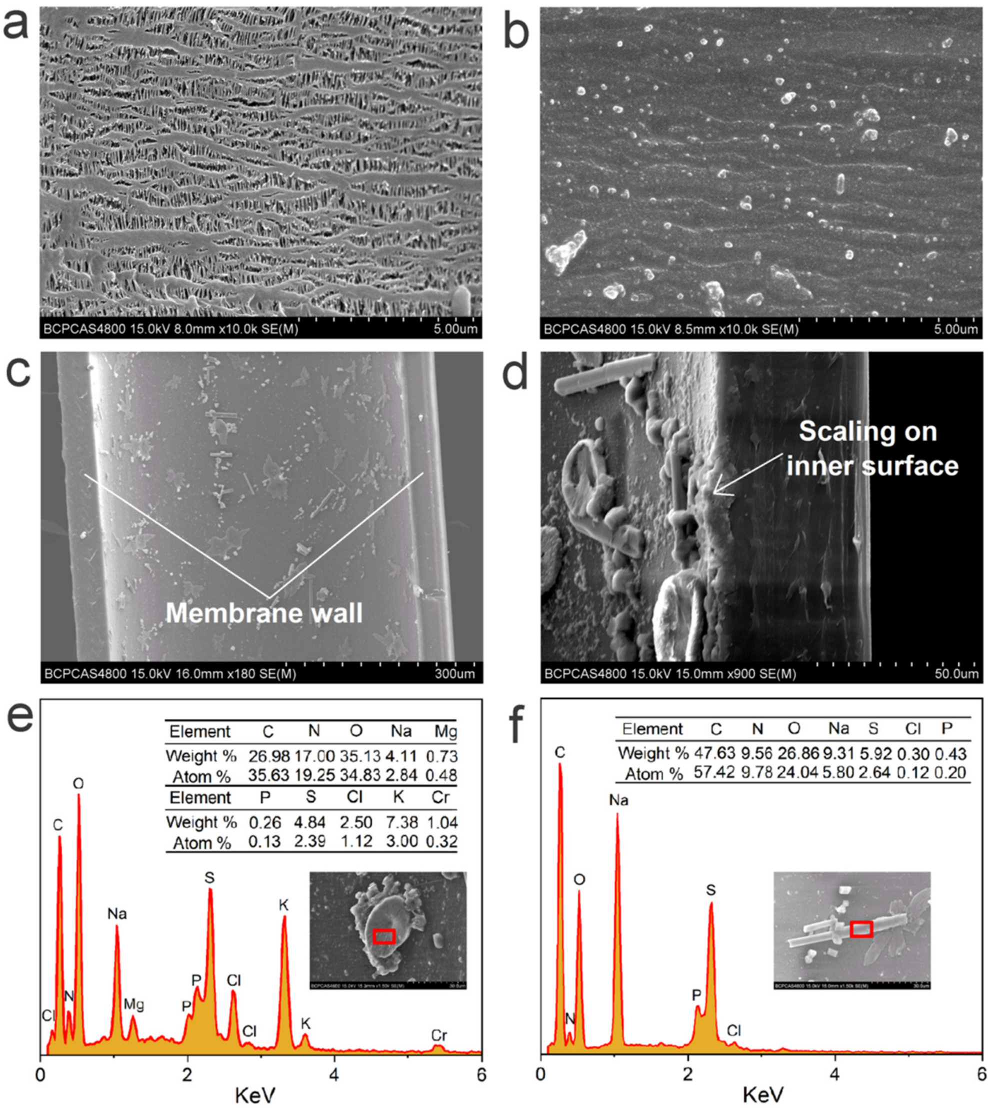

Figure S4. Morphological and elemental analyses of membrane samples after 91% water recovery. SEM images of the membrane on outer surface (a) and inner surface (b); representative morphology of the fouling layer on the inner surface (c) and a cross section (d); elemental analysis of a typical region in the fouling layer (e, f).

S8 Image of the membrane modules after 30 batches of VMD testing (a) and local enlarged image of the fouled membrane (b)

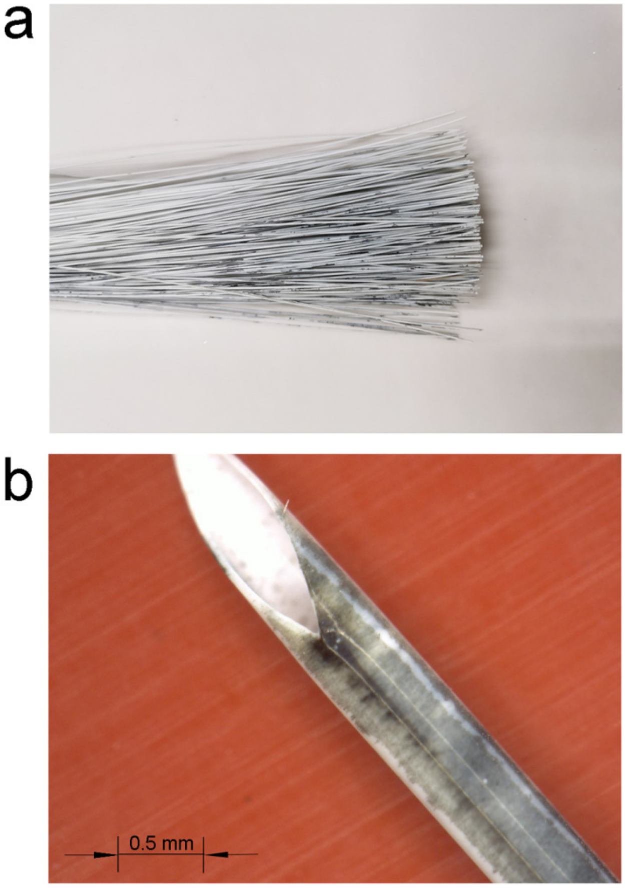

Figure S5. Image of the membrane modules after 30 batches of VMD testing (a) and local enlarged image of the fouled membrane (b).

S9 Water contact angle change images of the virgin (a–d) and used (e–h, at the fouled position) PP hollow fiber membrane

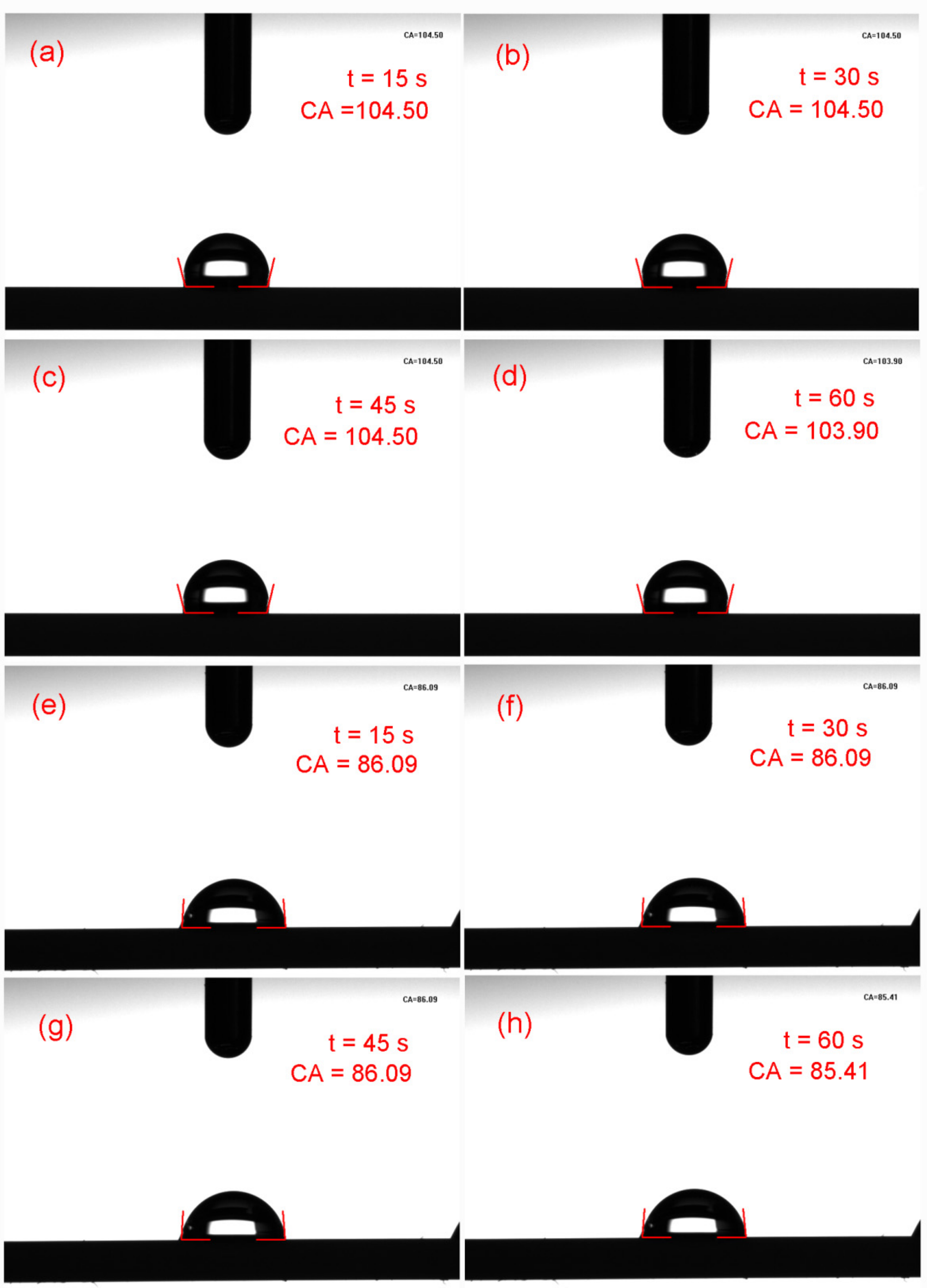

Figure S6. Water contact angle change images of the virgin (a–d) and used (e–h, at the fouled position) PP hollow fiber membrane.

References

1. Abu-Zeid, M. A. E.-R.;Zhang, Y.;Dong, H.;Zhang, L.;Chen, H.-L.;Hou, L. A comprehensive review of vacuum membrane distillation technique. Desalination **2015**, 356, 1-14.
2. Shao, H.;Wei, F.;Wu, B.;Zhang, K.;Yao, Y.;Liang, S.;Qin, S. Effects of annealing stress field on the structure and properties of polypropylene hollow fiber membranes made by stretching. RSC Advances **2016**, 6, 4271-4279.
3. Saffar, A.; Carreau, P. J.; Ajji, A.; Kamal, M. R. Influence of Stretching on the Performance of Polypropylene-Based Microporous Membranes. Industrial & Engineering Chemistry Research **2014**, 53(36):14014-14021.
4. Zhang, J.;Li, J.-D.;Duke, M.;Hoang, M.;Xie, Z.;Groth, A.;Tun, C.;Gray, S. Modelling of vacuum membrane distillation. J. Memb. Sci. **2013**, 434, 1-9.
5. Alkhudhiri, A.;Darwish, N.;Hilal, N. Membrane distillation: A comprehensive review. Desalination **2012**, 287, 2-18.
6. Zhang, J.;Li, J.-D.;Duke, M.;Xie, Z.;Gray, S. Performance of asymmetric hollow fibre membranes in membrane distillation under various configurations and vacuum enhancement. J. Memb. Sci. **2010**, 362, 517-528.
7. Ashoor, B. B.;Mansour, S.;Giwa, A.;Dufour, V.;Hasan, S. W. Principles and applications of direct contact membrane distillation (DCMD): A comprehensive review. Desalination, **2016** 398, 222-246.
8. El-Bourawi, M. S.; Khayet, M.; Ma, R.; Ding, Z.; Li, Z.; Zhang, X. Application of vacuum membrane distillation for ammonia removal. J. Memb. Sci. **2007**, 301, 200-209.
9. Mohammadi, T.;Kazemi, P. Taguchi optimization approach for phenolic wastewater treatment by vacuum membrane distillation. Desalination and Water Treatment **2013**, 52, 1341-1349.
10. Binns, E. H . The dissociation constant of phenol in water between 25°C and 60°C. Transactions of the Faraday Society **1959**, 55:1900-1903.
